# Analysis of the joint effect of SNPs to identify independent loci and allelic heterogeneity in schizophrenia GWAS data

**DOI:** 10.1038/s41398-017-0033-2

**Published:** 2017-12-18

**Authors:** Tatiana Polushina, Sudheer Giddaluru, Francesco Bettella, Thomas Espeseth, Astri J. Lundervold, Srdjan Djurovic, Sven Cichon, Per Hoffmann, Markus M. Nöthen, Vidar M. Steen, Ole A. Andreassen, Stéphanie Le Hellard

**Affiliations:** 1 NORMENT-K.G. Jebsen Center for Psychosis Research, Department of Clinical Science, University of Bergen, Bergen, Norway; 2 Dr. Einar Martens Research Group for Biological Psychiatry, Center for Medical Genetics and Molecular Medicine, Haukeland University Hospital, Bergen, Norway; 3 NORMENT-K.G. Jebsen Center for Psychosis Research, Institute of Clinical Medicine, University of Oslo, Oslo, Norway; 4 NORMENT-K.G. Jebsen Centre for Psychosis Research, Division of Mental Health and Addiction, Oslo University Hospital, Oslo, Norway; 5 Department of Psychology, University of Oslo, Oslo, Norway; 6 K.G. Jebsen Centre for Research on Neuropsychiatric Disorders, University of Bergen, Bergen, Norway; 7 Department of Biological and Medical Psychology, University of Bergen, Bergen, Norway; 8 Department of Biomedicine, Division of Medical Genetics, University of Basel, Basel, Switzerland; 9 Department of Genomics, Life and Brain Center, University of Bonn, Bonn, Germany; 10 Institute of Human Genetics, University of Bonn, Bonn, Germany; 11 Institute of Neuroscience and Medicine (INM-1), Research Center Juelich, Juelich, Germany

## Abstract

We have tested published methods for capturing allelic heterogeneity and identifying loci of joint effects to uncover more of the “hidden heritability” of schizophrenia (SCZ). We used two tools, cojo-GCTA and multi-SNP, to analyze meta-statistics from the latest genome-wide association study (GWAS) on SCZ by the Psychiatric Genomics Consortium (PGC). Stepwise regression on markers with *p* values <10^−7^ in cojo-GCTA identified 96 independent signals. Eighty-five passed the genome-wide significance threshold. Cross-validation of cojo-GCTA by CLUMP was 76%, i.e., 26 of the loci identified by the PGC using CLUMP were found to be dependent on another locus by cojo-GCTA. The overlap between cojo-GCTA and multi-SNP was better (up to 92%). Three markers reached genome-wide significance (5 × 10^−8^) in a joint effect model. In addition, two loci showed possible allelic heterogeneity within 1-Mb genomic regions, while CLUMP analysis had identified 16 such regions. Cojo-GCTA identified fewer independent loci than CLUMP and seems to be more conservative, probably because it accounts for long-range LD and interaction effects between markers. These findings also explain why fewer loci with possible allelic heterogeneity remained significant after cojo-GCTA analysis. With multi-SNP, 86 markers were selected at the threshold 10^−7^. Multi-SNP identifies fewer independent signals, due to splitting of the data and use of smaller samples. We recommend that cojo-GCTA and multi-SNP are used for post-GWAS analysis of all traits to call independent loci. We conclude that only a few loci in SCZ show joint effects or allelic heterogeneity, but this could be due to lack of power for that data set.

## Introduction

Schizophrenia (SCZ) places a heavy burden on patients and on society. Its heritability is estimated by recent twin or family studies to be between 64^1^ and 75%^2^. Genome-wide association studies (GWAS) have contributed important information about genetic markers of the disorder; the most recent Psychiatric Genomics Consortium (PGC) meta-analysis identified 108 genomic loci containing common genetic variants associated with SCZ^3^. However, each of these common genetic factors has only a small effect on the disease susceptibility. The relatively low proportion of variance explained by genome-wide-significant hits from GWAS data is a typical observation for complex traits with polygenic architecture.

In classical GWAS analysis, the collection of signals with an association *p* value below the genome-wide threshold (5 × 10^−8^) constitutes the set of associated loci. Although this cutoff is necessary to avoid type 1 errors, it has been shown since such a conservative approach probably creates many type 2 errors, leaving numerous associations of smaller effect undetected. Purcell et al.^4^ first showed that with increasingly liberal significance thresholds, more variability in complex disorders, including SCZ, could be explained. The proportion of variance in case–control status that can be explained by the genotyped single-nucleotide polymorphisms (SNPs) significantly increases when the threshold is lowered from the genome-wide significance level to 0.05 for each of 40 target subgroups of the primary GWAS (for example, for the Edinburgh cohort it increases from 0.027 to 0.286)^3^. Across all the samples, the estimated variance rises from 0.026 to 0.184. The polygenic risk score calculated using the significance threshold of 0.05 explains nearly 7% of variance on the liability scale across all the samples, whereas the genome-wide significant hits only explain about 3.4% of variance. This difference between the phenotypic variance explained by genome-wide significant SNPs and the phenotypic variance explained by genotyped variation is well-documented and known as “hidden heritability.” It will probably become smaller as larger samples are analyzed, but alternative statistical methods may also help to capture some of this association signal. For instance, joint effects tools focus on allelic heterogeneity and imperfect tagging data^5,6^. In the case of imperfect tagging, a single genotyped (or imputed) variant might not entirely explain the variation at a locus that occurs due to a single unknown causal variant. In the case of allelic heterogeneity, a single hit is unlikely to capture all the linkage disequilibrium (LD) between the several unknown causal SNPs and the genotyped variants at the locus. For example, the locus may contain two causal variants, the first increasing the risk for a trait, and the second one being protective. If these markers are correlated, marginal effect methods cannot detect the associations, because individuals who carry both variants have very little or no increased risk for a disorder. For those situations, a model where observed phenotype is influenced at each locus by variants that could be approximated by a linear combination of several independent observed markers is more reasonable and would explain more phenotypic variation. Using joint and conditional (cojo-GCTA) analysis of GWAS data for height, Yang et al.^5^ identified loci with multiple independent SNPs, and found 49 additional associated SNPs that explained around 1.3% of the phenotypic variation. Using the multi-SNP approach, Ehret et al.^6^ showed that 3, 2, and 1% of additional phenotypic variance could be explained for height, body mass index, and waist-to-hip ratio, respectively.

Our goal is to better understand the complex genetic architecture of SCZ using post-GWAS analysis tools. One of the most common approaches is to apply conditional regression within the locus of interest. However, this requires genotype data that is not available for meta-statistics. Cojo-GCTA and multi-SNP are the only methods with robust approximations for conditional regression analysis of summary data. We therefore applied these two statistical methods to the meta-statistics from the latest PGC SCZ GWAS (35,476 cases and 46,839 controls^3^) to test whether they can identify loci with joint effects.

## Subjects and methods

### Participant samples

The PGC performed a meta-analysis of GWAS data in a discovery set with 35,476 cases and 46,839 controls from 46 cohorts of European ancestry, 3 cohorts of East Asian ancestry, and 3 family-based samples of European ancestry, including 1235 parent affected-offspring trios^3^. Since we planned to perform a comparison with the PCG-SCZ CLUMP analysis, we used the quality control (QC) protocol (Supplementary Methods) from the PGC-SCZ paper, retaining a final set of 3,485,365 SNPs^3^.

### Norwegian and German LD reference samples

The Norwegian reference sample comprised individual genotypes from the Norwegian Cognitive NeuroGenetics sample (NCNG, *N* = 670)^7^ and the Norwegian Thematically Organized Psychosis sample (TOP, *N* = 1578)^8^. QC for each of these cohorts was performed with the PLINK tool^9^. Samples were excluded based on heterogeneity, relatedness, and call rate. Parameters for QC were: HWE *p* value of <1 × 10^−3^; minor allele frequency of <0.01; missingness 0.05. SNP data for the NCNG and TOP cohorts were imputed to the 1000 Genomes panel by MACH^10^ separately and merged after QC.

The German reference samples comprised individual genotypes from the Heinz Nixdorf RECALL study^11^. Genotyping was performed on the HNR-HumanOmniExpress_12v1_B, HNR-HumanOmniExpress_12v1_H, and HNR-HumanOmni1-Quad_v1_H chips. The QC parameters for retaining SNPs and subjects were: SNP missingness <0.05 (before sample removal); subject missingness <0.05; HWE *p* value >10^−4^. In addition, only individuals with concordant sex information were retained, and only one subject was kept for each pair of individuals with $\hat \pi$ > 0.1875. The processed samples were merged and imputed to the 1000 Genomes panel using the ENIGMA imputation protocol^12,13^.

The imputed Norwegian and German samples were both checked for HWE *p* values, imputation score, missingness, and outliers. After stringent QC (Supplementary Methods), the number of unrelated individuals in the Norwegian and German cohorts was 2200 and 2478, respectively. For the conditional analysis, we kept 7,111,233 markers that were present in both the Norwegian and German genotypes.

### Statistical analyses with cojo-GCTA

In this model^5^, each locus is interrogated with a joint combination of several independent markers. The approximate LD structure is obtained from an external reference sample, and the SNPs are selected in a stepwise manner using the GCTA tool presented by Yang et al.^5^ For replication, the authors^5^ recommend performing the joint analysis with two reference populations that are independent from each other, each containing >2000 unrelated individuals to avoid bias. We conducted the stepwise procedure in GCTA with various thresholds from 10^−3^ to 5 × 10^−8^ and two external LD reference samples, the Norwegian and German cohorts. In this step, the Norwegian LD reference sample was used for identification of SNPs, and the German LD reference sample was used for validation of the findings. We observed that below the threshold of 10^−7^, the validation rate drops substantially. Therefore, we selected the threshold of 10^−7^ for the main analysis with the merged Norwegian and German samples as the LD reference.

### Validation with multi-SNP

Since we wanted to further validate the results from cojo-GCTA, we also compared the list of independent SNPs identified by cojo-GCTA with the list of loci of joint effect identified by the multi-SNP method^6^. Both methods apply joint effect models, but the validation methods vary. Ehret et al.^6^ recommend splitting the sample into discovery and validation subsets to avoid bias in the SNP selection process. The discovery subset is used to extract a set of markers that (1) show significant association in the discovery subset (*p* value *<*10^−7^) and (2) are not in pairwise LD (*r* ^2^ > 0.1) with any other markers with the lowest *p* value. The replication subset is used for unbiased estimation of the effect sizes for the selected multi-SNP list. In this step, the tool performs estimation of the joint effect of the multi-SNP list.

We applied the multi-SNP association method to the summary statistics from the PGC SCZ subsets. We obtained access to summary statistics for 52 individual subsamples and randomly split the subsamples into discovery and validation sets. Using the METAL tool^14^, we performed meta-analysis on the summary statistics from 26 individual subsamples as the discovery set and on the summary statistics from 26 individual subsamples as the validation set. We kept the same 3,485,365 SNPs in both sets that were analyzed by cojo-GCTA. The European cohort from the HapMap project release II + III^15^ was used to estimate the LD structure^6^.

### Gene annotation

The final list of SNPs that showed joint effects and genome-wide significant *p* values after cojo-GCTA, and which had not been reported previously by the PGC-SCZ, was used to define new genomic loci associated with SCZ. The genomic loci were defined by the associated SNP and all SNPs in LD with the associated SNP (*r* ^*2*^ > 0.2). Gene annotations were performed with the aid of the LDsnpR package^16^ and the RefSeq gene list^17^.

### Code availability

The following programs were used in this study: GCTA (cojo-GCTA option)^5^; multi-SNP^6^; PLINK^9^; MACH^10^; METAL^14^; and LDSnpR^16^. All are publicly available.

## Results

We applied conditional regression-GCTA (cojo-GCTA)^5^ and multi-SNP^6^ to the PGC-SCZ GWAS summary data. In these two methods, each locus is analyzed with a combination of several independent markers, corrected for LD between the markers.

### Identification of an independent set of SNPs associated with SCZ using cojo-GCTA

Cojo-GCTA requires summary statistics (effect size, standard error, *p* value, and allele frequency) and genotypes from a reference population for LD estimation. We used about 3.5 million SNPs from the publicly available PGC-SCZ summary statistics^3^. Since the LD structure of the major histocompatibility complex (MHC) region on chromosome 6 is challenging for “multi-SNP” analysis and requires specific analyses based on genotypes, we chose to exclude this region from our study. In the PGC SCZ paper^3^, the MHC is represented only by the single most significant SNP. We used a Norwegian sample (*N* = 2200 after QC) for LD reference, and an independent reference sample of German origin (*N* = 2478) for validation.

The stepwise conditional regression implemented in cojo-GCTA corrects *β* and *p* values of neighboring SNPs (in a sliding window of 10 Mb) based on the LD between the SNPs. This stepwise procedure ensures that the SNP with the lowest *p* value is selected first for conditioning the effect on neighboring loci based on the LD between the neighboring SNPs and the selected SNP. Following LD-based correction of effect, all SNPs that remained significant under a fixed threshold are run through the same process in a stepwise manner. This process identifies (1) the number of independent signals in a region, and (2) association signals due to the joint effect of several SNPs. By lowering the threshold for cojo-GCTA, we can include more SNPs in the analysis and potentially identify more loci with joint effects that failed to be identified by single-marker analysis. We still keep the genome-wide significance threshold to call associated loci, whether they are due to a single effect or to a joint effect.

We first tested the effect of lowering the significance threshold for the stepwise regression on the level of validation when the Norwegian or German samples were used for LD reference (Table 1). When the threshold was lowered from 10^−3^ to 5 × 10^−8^, several SNPs became significant at the genome-wide significance threshold due to the joint effects of neighboring markers. The SNPs identified using the Norwegian LD reference sample were then tested for validation using the German LD reference sample. SNPs were considered to be validated if the joint *p* values passed the genome-wide significance threshold (5 × 10^−8^) with both LD references and if the joint *p* values did not change between the two LD references. The procedure is summarized in Supplementary Fig. 1.

**Table 1:** Number of independent SNPs for different thresholds

Threshold *p*_th_	10^−3^	10^−4^	10^−5^	10^−6^	10^−7^	5 × 10^−8^
Number of markers below threshold before CR^a^	82,041	35,630	18,710	11,028	6533	5706
Number of markers below threshold after CR^b^	7038	1048	460	200	101	88
Number of markers below 5 × 10^−8^ after CR^c^	2504	186	134	92	90	88
Number of markers validated with German cohort as LD reference^d^	—	69	74	80	83	83
% Markers validated with German cohort as LD reference^e^		37%	55%	87%	92%	94%

*CR* conditional regression, *LD* linkage disequilibrium, *p* *Germ joint* *p* values with German LD reference, *p* *Norg joint* *p* values with Norwegian LD reference

^a^Number of SNPs below the indicated threshold in the initial data set

^b^Number of markers that were selected using the stepwise procedure with the Norwegian LD reference sample

^c^Number of signals that passed the genome-wide significance threshold in the joint model

^d^Number of validated SNPs: a marker is deemed validated for joint effect if it passes the genome-wide significance level (5 × 10^−8^) after stepwise analysis with the Norwegian LD reference sample and after joint analysis with the German LD reference sample, and if −log_10_(*p*Germ)/−log_10_(*p*Norg) <2 (the joint *p* values estimated using the Norwegian sample as LD reference do not differ essentially from the joint *p* values estimated using the German LD reference)

^e^Percentages of validated SNPs for different thresholds. For the threshold 10^−3^, the list of selected markers after the stepwise procedure could not be fitted with the German sample because of redundant signals

By decreasing the threshold to <10^−7^, we observed that the increase in additional loci identified by joint effects in the Norwegian sample did not lead to an increase in loci validated using the German sample as LD reference. This is probably because by lowering the significance threshold, we identified more signals due to population-specific LD patterns.

The allele frequencies of the selected SNPs at the threshold 10^−7^ estimated from either the Norwegian or the German cohort were concordant with the frequencies for the list used for the PGC-SCZ data (Supplementary Fig. 2).

Thus, we established the optimal threshold to perform the stepwise conditional regression on all SNPs with *p* value <10^−7^, and we set 5 × 10^−8^ as the significance threshold for the combined effect *p* value after conditional regression. In addition, after establishing those thresholds, we used the merged German and Norwegian samples (*N* = 4628, Supplementary Fig. 3) to apply cojo-GCTA to the PGC-SCZ summary statistics to identify loci showing allelic heterogeneity and joint effects. This better reflects the mixed European populations used in the PGC-SCZ, and thus lowers the risk of spurious hits while also increasing the accuracy of joint effect estimation^5^.

Stepwise regression identified 96 independent markers associated at the 10^−7^ threshold. Eighty-five of them passed the genome-wide significance level (Supplementary Table 1). The other loci, with a joint *p* value <10^−7^ but >5 × 10^−8^, are presented in Supplementary Table 2.

Next, we compared our results with independent signals identified in the publicly available PGC-SCZ discovery data (*N* = 82,315) using CLUMP^3,9^. A total of 108 independent SNPs were identified as significant by the PGC-SCZ CLUMP analysis (Supplementary Methods). In the PGC study, the strongest associated SNP, rs114541829 (*p* value of 3.48 × 10^−31^), represents the MHC region. For comparison with the cojo-GCTA results, we excluded this SNP and used the remaining 107 markers.

Of the 85 independent markers identified by cojo-GCTA, 81 were matched to genome-wide significant SNPs in the CLUMP PGC-SCZ data. The matching cojo-GCTA markers were either exactly the same as, or in LD with, the CLUMP PGC-SCZ SNPs. Of the 107 CLUMP PGC-SCZ markers, 26 were not significant after cojo-GCTA. A major reason for this difference is that cojo-GCTA uses a 10 Mb window for LD estimation, whereas CLUMP was performed with a 500 kb window. From the original study^5^, for an LD window of 10 Mb or larger, the observed LD correlation between SNPs does not differ substantially from that expected by chance. Supplementary Table 1 shows that the *p* value of all 26 markers identified by CLUMP but not cojo-GCTA was reduced after conditioning to another associated SNP lying within a 10 Mb window. If we use a 500 kb window, cojo-GCTA selects 101 signals that passed the genome-wide significance threshold. We compared these 101 SNPs with those identified by CLUMP and by cojo-GCTA with a 10 Mb window (Supplementary Methods and Supplementary Table 3). We found that cojo-GCTA with a 10 Mb window produces more stable results. At the same time, it accounts for joint effects in long-range LD, which cannot be estimated by CLUMP.

### Identification of loci with joint effects

Out of the 85 significant independent SNPs identified by cojo-GCTA (Supplementary Table 1 **)**, four did not reach significance in the PGC-SCZ discovery sample (Table 2). One of these markers became significant in the replication and discovery samples tested in the PGC-SCZ study, when an additional sample of 1513 cases and 66,236 controls was included in the meta-analysis. This highlights that in some cases, cojo-GCTA can increase the power to detect additional association signals without having to increase the sample size.

**Table 2:** SNPs that became significant in the joint analysis using the merged Norwegian and German cohorts as LD reference

SNP^a^	Position^b^	*p* value PGC discovery^c^	pJ after CR^d^	PGC *p* value discovery + replication^e^
rs1509378	2: 22,754,466	8.37 × 10^−8^	4.23 × 10^−8^	Not reported
rs12474906	2: 28,033,538	1.01 × 10^−7^	4.99 × 10^−8^	1.36 × 10^−7^
rs12148337	15: 70,589,272	5.33 × 10^−8^	6.51 × 10^−9^	1.78 × 10^−8^
rs2398180	15: 96,863,169	0.002	6.37 × 10^−9^	Not reported

^a^dbSNP reference ID for the SNP

^b^Genomic position (chromosome:base pair) of the marker based on UCSC hg19/NCBI build 37

^c^ *p* values in the PGC-SCZ discovery sample

^d^ *p* values in the joint effect model with merged Norwegian and German LD reference samples

^e^Information from the PGC for replication testing of each marker

In addition, SNPs in three loci showed increased significance after joint model analysis (Supplementary Table 1). Two of these loci are on chromosome 15. The significance level of the corresponding markers, rs950169 (7.62 × 10^−11^) and rs4702 (2.30 × 10^−12^), substantially increased to 5.44 × 10^−18^ and 2.06 × 10^−24^ respectively, after combined analysis. The third locus, on chromosome 22, contains the signals rs61298040 (3.90 × 10^−8^) and rs1023500 (5.04 × 10^−8^). The significance levels of these two markers were improved to 4.02 × 10^−12^ and 5.59 × 10^−12^, respectively.

In the original cojo-GCTA article, the authors compared the proportion of explained phenotypic variance based on only the strongest signal per locus or based on multiple signals after running the joint model. They showed that by including additional markers, the total explained phenotypic variance increases. We were unable to test for the increase in variance explained by these joint effects because we do not have access to the genotypes and case–control status of the samples.

### Identification of two loci with allelic heterogeneity

**Fig. 1:**
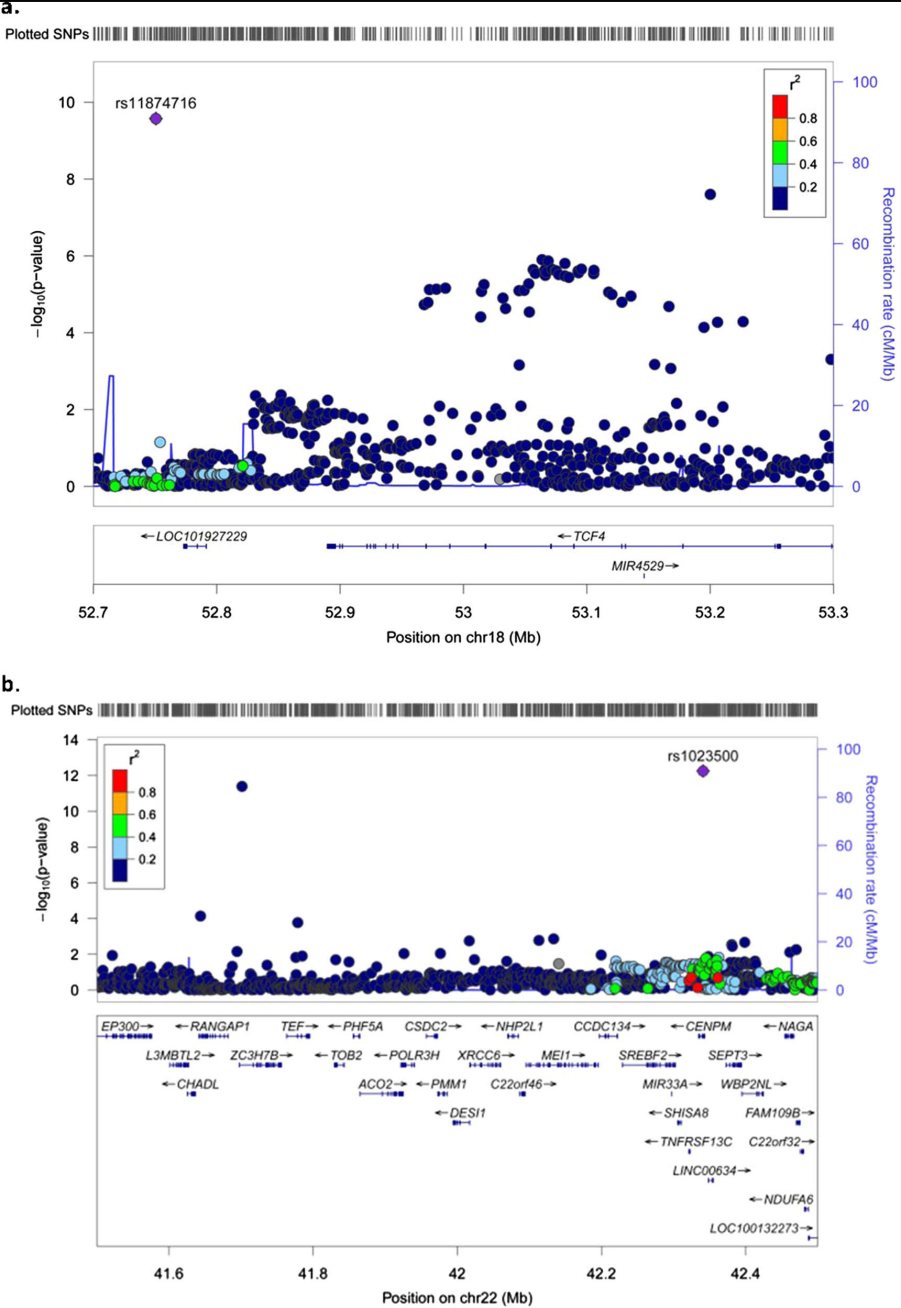
Loci with multiple independent signals of association The plots show the results for the joint and conditional analysis with merged Norwegian and German cohorts as the LD reference sample for the loci on chromosome 18 **(a)** and chromosome 22 **(b)**. On each plot, several independent signals are identified using the stepwise procedure within a 10 Mb window in cojo-GCTA. SNPs are plotted according to their chromosomal positions based on UCSC hg19/NCBI build 37. The –log_10_(*p* values) of the SNPs are shown on each plot. LD values between the lead SNP and the other markers are indicated by color. Genes located in the region of interest are indicated at the bottom. Plots were generated using the LocusZoom tool^34^

Most of the genomic regions identified by cojo-GCTA contained only one signal of association when a genomic window of 1 Mb was examined around the association. However, we found two regions that contained several independent signals within <1 Mb (Fig. 1a). One region, on chromosome 18, contains two independent SNPs, rs11874716, and rs9636107, with joint *p* values of 8.67 × 10^−12^ and 3.73 × 10^−8^, respectively. This region covers 449 kb and contains just one gene, *TCF4* ^18^. The second region (Fig. 1b) contains two independent markers, rs61298040 and rs1023500, with joint *p* values of 4.02 × 10^−12^ and 5.59 × 10^−13^. This 640 kb region on chromosome 22 contains 18 genes. Using the same 1 Mb window criterion, 16 loci with potential allelic heterogeneity were identified by CLUMP (Supplementary Table 1). Thus, it seems that cojo-GCTA is also more conservative in its estimates than the CLUMP procedure in assessing allelic heterogeneity.

### Annotation of loci

We annotated only the regions that became significant in cojo-GCTA at the genome-wide significance level, and that were not described previously by the PGC-SCZ study. For these three regions of association (Table 3), the genomic boundaries were defined based on the LD around the associated markers. Two genes mapped to a genomic region on chromosome 15 that contains the genes *NR2F2* and *NR2F2-AS1* (Supplementary Table 4). These two genes were previously associated with migraine and motion sickness^19,20^. Ten genes mapped to a locus on chromosome 2 containing the genes *C2orf16*, *ZNF512*, *CCDC121*, *GPN1*, *SUPT7L*, *SLC4A1AP*, *MRPL33*, *RBKS*, *BRE*, and *MIR4263* (Supplementary Table 4). *SLC4A1AP* has been associated with Alzheimer’s disease^21^. *C2orf16* and *ZNF512* are related to triglyceride and calcium levels^22–24^. In the third genomic locus, also on chromosome 2, the marker rs1509378 maps to *LOC102723362*, which is associated with self-rated health and low-density lipoprotein cholesterol level^25,26^ (Supplementary Table 4).

**Table 3:** Annotation of the independent markers to genes

Position^a^	Gene(s) in region^b^
2: 22,621,296–22,821,666	*LOC102723362*
2: 27,784,034–28,281,545	*C2orf16*, *ZNF512*, *CCDC121*, *GPN1*, *SUPT7L*, *SLC4A1AP*, *MRPL33*, *RBKS*, *BRE*, *MIR4263*
15: 96,817,467–96,866,320	*NR2F2*, *NR2F2-AS1*

^a^Locus positions are displayed as chromosome:start–end based on UCSC hg19/NCBI build 37. Loci were delimited by taking into account all markers in LD with the marker selected by cojo-GCTA

^b^Regions were screened for gene content using RefSeq in the UCSC genome browser

### Validation of findings with multi-SNP

We next analyzed the same GWAS summary statistics with multi-SNP. This method^6^ filters markers based on their marginal effects and *p* values and pairwise LD. The authors recommend splitting the meta-statistics into discovery and validation subsets to avoid any bias in estimations^6^. Thus, we performed a discovery meta-analysis of 26 single sample summary statistics and a validation meta-analysis of 26 single sample summary statistics. Markers were declared associated if they were identified in the discovery sample and validated in the replication sample at 10^−7^ after running multi-SNP. For this procedure, the LD window was 1 Mb and the *r* ^*2*^ threshold was 0.1 (HapMap 2 CEU reference sample). A total of 86 loci were identified.

We then compared the 86 multi-SNP SNPs with the 96 cojo-GCTA markers with joint *p*_j_ value <10^−7^. As many as 84 of the 86 multi-SNP loci corresponded to loci identified by the cojo-GCTA procedure, i.e., 87% of the loci identified by cojo-GCTA are validated by multi-SNP (Supplementary Table 5). Remarkably, if we consider only the 85 loci that reach genome-wide significance after cojo-GCTA, 79 of them overlap with the multi-SNP loci (i.e., 92% validation). Two markers identified by multi-SNP, rs10860964, and rs2434531, did not reach the significance threshold after cojo-GCTA analysis (Supplementary Table 5). Two markers identified by multi-SNP (rs1451488 and rs12996313) corresponded to one marker identified by cojo-GCTA (rs796364). Out of the 84 regions of association identified by multi-SNP, 74 SNPs corresponded to either the same SNPs identified by cojo-GCTA or to markers in strong LD. The remaining nine markers are located within 1 Mb of the genomic loci identified by cojo-GCTA.

The additional 13 regions identified with cojo-GCTA but not with multi-SNP analysis are most likely explained by differences in (1) the external LD reference (since cojo-GCTA uses a German/Norwegian genotyped sample, whereas multi-SNP uses the HapMap 2 CEU reference sample^11^), and (2) the statistical method used to calculate the joint effects and the power of the studies, since the multi-SNP meta *p* values were calculated using smaller discovery and validation sets.

## Discussion

In this study, we have used the conditional regression method from Yang et al.^5^ (cojo-GCTA) and the multi-SNP method from Ehret et al.^6^ on the meta-statistics from the latest analysis of SCZ by the PGC^3^, to try and capture allelic heterogeneity and loci of joint effects in this GWAS. At present, there is no “gold standard” for conditional regression analysis of metastatistics, but these approaches have successfully identified additional independent signals in GWAS of height (*N* = 49 in the paper by Yang et al.^5^ and *N* = 44 in the paper by Ehret et al.^6^). In the body mass index (BMI) GWAS, Yang et al.^5^ did not find additional SNPs, while Ehret et al.^6^ found 10 new signals. These results suggest that the portion of the missing heritability due to multiple independent effects per locus is not insignificant, but varies across human traits, which might be due to different polygenicity level or to power.

Using the strict threshold of *p* value <10^−7^ to select SNPs in stepwise regression, cojo-GCTA identified 96 independent signals, 85 of which passed the genome-wide significance threshold. The PGC-SCZ study, using the CLUMP method, identified 107 SNPs (excluding the MHC region) in independent loci in the discovery sample. One reason for this difference is that the two methods correct for LD and assess the range of LD in different ways. For a single locus, cojo-GCTA can account for longer range LD effects up to 10 Mb, while CLUMP has so far been limited to 500 kb. Therefore, with cojo-GCTA, we can correct for LD effects in larger regions. The other main difference is that cojo-GCTA adjusts the *β* values of neighboring markers, thus taking into consideration both LD and the direction of effects between SNPs. While the validation of cojo-GCTA by multi-SNP was up to 92%, the validation of cojo-GCTA by CLUMP was 76%. Taking these findings into consideration, it appears that the set of independent SNPs identified by cojo-GCTA is more conservative than that identified by CLUMP. Thus, in future studies and in analyses of independent markers in GWAS, we would recommend applying the cojo-GCTA and/or multi-SNP methods as complementary approaches to the standard CLUMP procedure, since the tools are easy, versatile, and computationally fast. Both methods have shown good agreement with each other and with the CLUMP analysis. In contrast to cojo-GCTA, the multi-SNP protocol suggests splitting the summary statistics into discovery and validation sets. This avoids selection bias, but at the same time, the sample sizes are decreased, and therefore there is less power to identify signals of association^27^. These secondary analyses of GWAS were performed recently with cojo-GCTA for several phenotypes (coronary artery disease^28^, educational attainment^29^, subjective well-being, depressive symptoms, and neuroticism^30^), and will most likely become more common in future GWASs.

After including cojo-GCTA, four additional SNPs became significant in the joint effect model, and the level of significance of three of them increased substantially. The significance level was unchanged for the majority of independent markers. Applying the same method to a GWAS of height, Yang et al.^5^ found that the significance of 29 SNPs (out of a total of 247) was greatly improved, while 2 SNPs for BMI were improved at the threshold 5 × 10^−6^ and none at the genome-wide significance level. Similarly, the number of loci with multiple independent SCZ-associated SNPs was relatively low, i.e., only two 1 Mb loci had possible allelic heterogeneity. In comparison, Yang et al.^5^ identified 36 loci showing allelic heterogeneity for height in the GIANT samples, but none for BMI. We have provided comparison of cojo-GCTA results across several traits with complex polygenic architecture in the Supplementary Material and conclude that the density of markers is unlikely to explain the difference seen in the number of loci with joint effects or allelic heterogeneity between GWAS. The number of independent markers probably depends on the power of the initial GWAS. Thus, it will be interesting to perform cojo-GCTA in studies of larger SCZ samples, to evaluate how much allelic heterogeneity is then found.

The two loci with evidence of allelic heterogeneity in SCZ are located on chromosomes 18 and 22. The locus on chromosome 18 encompasses the gene *TCF4*, which has previously been shown to have potential allelic heterogeneity, since other markers in the gene are associated with phenotypes related to SCZ^18^. The locus on chromosome 22 encompasses several genes and has been reported previously to be associated with SCZ^31^ and Alzheimer disease^32^. More work will be needed to understand the relationship between these independent markers and whether they target the same genes (especially for the locus on chromosome 22).

We excluded the MHC region from the analysis because this region presents a complex LD structure. Although conditional regression analyses have successfully identified the number of independent associations in other complex phenotypes associated with the MHC region, these analyses require access to genotypes, which were not available to us. Identifying the number of independent associations in the MHC was not the purpose of our investigation, and other studies have successfully focused specifically on this region. For instance, a recent study showed that the *C4* structure is crucial for the development of SCZ and might explain the main signal of association to this region in SCZ^33^.

Contrary to our expectations, we found that the number of markers showing genome-wide significance was lower when independent signals were identified with cojo-GCTA than with CLUMP, and we show that this is likely due to long-range LD. However, we confirm the association of 85 independent loci with SCZ (in addition to the MHC locus), and we identified two loci with multiple signals that should be further examined. Thus, the systematic analysis of independent markers located at the same loci with the methods we used here can enrich our current understanding of complex disease architecture and provide insights into designing further tools for post-GWAS studies.

## Electronic supplementary material

Supplementary Data

Supplementary Table 1

Supplementary Table 2

Supplementary Table 3

Supplementary Table 5
