# Supplementary material for: Analysis of the joint effect of SNPs to identify independent loci and allelic heterogeneity in schizophrenia GWAS data
Source: Transl Psychiatry. 2017 Dec 18;7:1289. doi: 10.1038/s41398-017-0033-2 (PMC5802566; doi:10.1038/s41398-017-0033-2)
Supplement: Supplementary file 1 — Supplementary Data [file 41398_2017_33_MOESM1_ESM.docx]

**SUPPLEMENTARY DATA**

Analysis of the Joint Effect of SNPs to Identify Independent Loci and Allelic Heterogeneity in Schizophrenia GWAS Data

Tatiana Polushina, Sudheer Giddaluru, Francesco Bettella, Thomas Espeseth, Astri J. Lundervold, Srdjan Djurovic, Sven Cichon, Per Hoffmann, Markus M. Nöthen, Vidar M. Steen, Ole A. Andreassen, Stéphanie Le Hellard.

**Page 2 Supplementary Methods**

**Page 7 Supplementary Tables**

**Page 11 Supplementary Figures**

**Page 15 Supplementary References**

**SUPPLEMENTARY METHODS**

***Participant Samples***

The PGC performed a meta-analysis of GWAS data in a discovery set with 35,476 cases and 46,839 controls (including 377 cases and 403 controls from the TOP study, Norway) (Ripke et al., 2014). Details of the quality control (QC) protocol and phenotypic characteristics are in the original paper (Ripke et al., 2014). Genotype data were imputed to 9.4 million SNPs present in the 1000 Genomes Project Phase 1 reference panel. We removed SNPs with imputation score <0.9, ambiguous SNPs, insertion/deletion SNPs, and SNPs with minor allele frequency (MAF) <0.10, retaining a final set of 3,485,365 SNPs. The allele frequencies were estimated based on the HapMap Phase 2, the CEU population.

From the PGC study, the locus with the strongest associated signal (p-value of 3.48×10^-31^) is the major histocompatibility complex (MHC) on chromosome 6. Analyzing the structure of the MHC region is especially challenging due to its complex LD. In the PGC study, only one SNP was kept to represent the entire region of 25-34 Mb. We excluded all the markers within a 10 Mb window around the MHC region, because they are conditioned on the strongest previously reported SNP.

***Norwegian LD Reference Samples***

The Norwegian reference samples comprised individual genotypes from the Norwegian Cognitive NeuroGenetics (NCNG, *n* = 670 (Espeseth et al., 2012)) and Norwegian Thematically Organized Psychosis (TOP, *n* = 1,578 (Athanasiu et al., 2010)) samples. Both cohorts are of Scandinavian origin with catchment in areas near Bergen (NCNG) and Oslo (TOP).

The NCNG samples were genotyped on the Illumina Human 610-Quad Beadchip. The initial NCNG GWAS consisted of 554,225 SNPs genotyped in a homogenous Norwegian sample of 670 individuals (213 males, 457 females). QC was performed with the PLINK tool (Purcell et al., 2007). Samples were excluded based on heterogeneity, relatedness, and call rate. Parameters for QC were: HWE p-value of <1×10^−3^, minor allele frequency (MAF) of <0.01, missingness 0.05. Pairwise genetic relationship between individuals was used to remove one of each pair of individuals with an estimated relatedness of >0.1. Population structure within the NCNG sample was examined with the multidimensional scaling algorithm (MDS) in PLINK and outliers were excluded (MDS PC3 <-0.03). After QC, 662 individuals and 544,905 SNPs were used for further analysis. The TOP sample was treated in the same way. After QC, 1,568 individuals were retained.

SNP data for the NCNG and TOP cohorts were imputed to the 1000 Genomes panel by MACH (Abecasis & Li, 2007). We used the best guess genotypes of the imputed SNPs and excluded imputed SNPs with HWE p-value of <1×10^−3^, imputation *r^2^* of <0.2 or MAF of <0.01 and missingness 0.05. The NCNG and TOP samples were then merged and checked again for quality (same parameters), giving a total of 7,529,100 SNPs in the Norwegian cohort. We repeated estimation of pairwise genetic relationship between individuals and removed one of each pair of individuals with an estimated relatedness of >0.1. We examined the population structure using MDS plots and excluded outliers by visually inspecting the MDS data. After QC, 2200 unrelated individuals were retained in the Norwegian cohort.

***German LD Reference Sample***

We obtained access to the individual-level genotypes of cohorts from the Heinz Nixdorf RECALL study (Schmermund et al., 2002). Samples were genotyped on the HNR-HumanOmniExpress_12v1_B, HNR-HumanOmniExpress_12v1_H and HNR-HumanOmni1-Quad_v1_H chips. The QC parameters for retaining SNPs and subjects were: SNP missingness <0.05 (before sample removal); subject missingness <0.05; SNP Hardy-Weinberg equilibrium p-value >10^−4^. In addition, only individuals with concordant sex information were retained, and only one subject was kept for each pair of individuals with $\hat{\pi}$ > 0.1875. The population structure of each sample was investigated with the MDS algorithm in PLINK, and outliers were excluded. The same QC protocol was applied for each of the three samples, and the processed samples were merged. The merged German GWAS consisted of 633,603 SNPs genotyped in a homogenous German sample of 2,509 individuals. After QC, 2,478 unrelated individuals were retained. SNP data for the German cohort were imputed to the 1000 Genomes panel using the ENIGMA imputation protocol (Stein et al., 2012; The Enhancing Neuroimaging Genetics through MetaAnalysis (ENIGMA) Consortium). After imputation, we applied a QC protocol with the following exclusion parameters: HWE p-value of <10^−6^; imputation *r^2^* of <0.2; MAF of <0.01; and missingness 0.05. Since exome genotypes were available for the German sample, we estimated the imputation accuracy to be around 0.97. The final QC protocol was identical to that used in the Norwegian sample. After QC, the German cohort consisted of 2,478 unrelated individuals with genotypes for 7,531,606 SNPs.

For conditional analysis we kept 7,111,233 markers that are present in both the Norwegian and German genotypes.

The population structures of the Norwegian sample and the German sample were compared with each other and with 11 different populations from HapMap3 using the multidimensional scaling algorithm (MDS) in PLINK (Supplementary Figure 3a). The German and Norwegian subjects are tightly clustered together with other European samples, indicating that most of the subjects were of European ancestry (Supplementary Figure 3b). The structural features of these reference cohorts should be similar to each other, and similar to the PGC samples, which include: European ancestry, 32,405 cases and 42,221 controls; Asian ancestry, 1,836 and 3,383 controls; and European ancestry trio samples, 1,235 pedigrees (Ripke et al., 2014). Therefore, the Norwegian and German samples were considered suitable as external reference samples for the estimated LD structure of the PGC data.

***CLUMP Paramaters***

We compared our results with the publicly available PGC-SCZ data, i.e. the discovery sample from the published study (*n* = 82,315). In this discovery sample, the CLUMP method (Purcell et al., 2007; Ripke et al., 2014) was used to identify independent loci associated at the genome-wide level. CLUMP removed SNPs within a 500 kb window that had an *r*^2^ ≥0.1 with another more significant marker. Markers with MAF <10% and imputation score <0.9 were also discarded. Using these criteria, 108 independent SNPs were identified as significant by the PGC-SCZ CLUMP analysis. For comparison with the cojo-GCTA results, we excluded SNP rs114541829 which represents the MHC region, and used the remaining 107 markers.

***Comparison of cojo-GCTA with a 10 Mb window, cojo-GCTA with a 500 kb window, and CLUMP***

In order to determine how much of the differences observed between cojo-GCTA and CLUMP were due to long-range LD effects, we compared the results obtained with cojo-GCTA run with a 500 kb window (cojo-GCTA-500), cojo-GCTA run with a 10 Mb window (cojo-GCTA-10M) and CLUMP.

With cojo-GCTA-500, we obtained 101 signals that passed the genome-wide significance threshold in the joint model. All of the 101 signals are related to the ones reported by the PGC using CLUMP, but the effects of the joint analysis varied across the signals (see Supplementary Table 3).

1. 60 of the signals were identified by all three approaches: CLUMP, cojo-GCTA-500 (joint analysis with a 500 kb window) and cojo-GCTA-10M (joint analysis with a 10 Mb window).
2. 3 signals identified by cojo-GCTA-10M are not significant in CLUMP and/or cojo-GCTA-500.
3. For 4 genomic loci, cojo-GCTA-10M identified one signal while both cojo-GCTA-500 and CLUMP identified more. The markers identified by CLUMP were either the same ones identified by cojo-GCTA-500 or different ones.
4. For two regions, cojo-GCTA-10M identified 2 signals while cojo-GCTA-500 and CLUMP identified 3.
5. For 3 regions, both cojo-GCTA-10M and CLUMP identified one independent signal, while cojo-GCTA-500 identified several.
6. Finally, for 10 regions, both cojo-GCTA-10M and cojo-GCTA-500 identified one signal while CLUMP identified several independent signals.

This highlights that the two methods (CLUMP and cojo-GCTA) provide complementary information due to how the LD information is integrated in the analysis (i.e. purely by removing SNPs correlated based on *r^2^* or by calculated joint effect). Considering the cojo-GCTA results only, the results obtained with the long-range window are more stable, while the shorter window overfits the model (see d). It is therefore preferential, as recommended by Yang et al., to use a 10 Mb window. Compared to CLUMP, cojo-GCTA-10M identifies better long-range LD effects, which are not possible to calculate with CLUMP (model limitation), as shown in points b) c) d) and f).

***Boundaries for Genomic Loci***

We annotated only the regions that became significant in cojo-GCTA at the genome-wide significance level, i.e the four regions displayed in Table 2. One of these regions was reported in the PGC meta-statistics because it became significant after replication. For the three other regions of association (Table 3), considering that each SNP in LD with the selected SNP is as good a candidate for functional effect as the one selected, we also included SNPs in LD ($r^{2}>0.2$) with the SNP selected during cojo-GCTA. Our final list contained 548 markers, which were used to define the boundaries of the three genomic regions.

***Imputation Density***

Here, we consider if the number of loci with joint effects or allelic heterogeneity might depend on the density of the markers genotyped and imputed in the different GWASs. The height and BMI phenotypes were analyzed in the same sample with a density of 2.5 million SNPs (Yang et al., 2012), while the SCZ GWAS in our study contained 3.5 million SNPs. In the case of BMI, there were no loci with allelic heterogeneity, whereas Yang et al (2012) identified 36 loci for height. In order to test the influence of imputation density, we have repeated the cojo-GCTA analysis for the PGC-SCZ meta-statistics, retaining the ones presented in the HapMap imputation panel, as was done for the Height and BMI GWASs. In that panel of 1,497,539 SNPs, 88 SNPs were selected as independent. Out of them, 3 became significant in the joint model, and there are only two examples of possible allelic heterogeneity. The cojo-GCTA tool selected fewer markers in this less dense GWAS set than in the more dense summary statistics, and all newly significant SNPs were the same or LD-linked with loci that we reported in the denser GWAS.

The low number of both heterogeneous loci and loci with joint effects in SCZ may reflect the limited power in the SCZ GWAS. The GIANT sample comprised 133,653 individuals for height and 123,865 for BMI, while the PGC-SCZ discovery sample is 82,315 individuals. In a GWAS of 185,000 individuals with coronary artery disease, cojo-GCTA identified twelve 1 Mb loci with multiple independent associated SNPs (Nikpay et al., 2015).

For complex traits with polygenic structure, the power of the study seems to play a more significant role than the marker density. For comparison, we have applied the same procedure to a number of studies with polygenic architecture: type 2 diabetes (Morris et al., 2012), harmonized neuroticism and extraversion (de Moor et al., 2015; van den Berg et al., 2014), and glucose level (Manning et al., 2012). For these traits, the sample sizes (69,033, 63,661 and 58,074, respectively) are not substantially different from that in the PGC-SCZ study. Due to lack of signal, only one independent SNP was selected in the harmonized neuroticism study and none for the extraversion trait. For type 2 diabetes, 9 independent signals were selected, but we have not observed any locus with multiple SNPs. For glucose level, 23 independent markers were selected, but none of the SNPs with p-values 5x10^-8^ < p-value < 10^-7^ improved the significance level. However, we observed two cases with multiple independent markers within a 1 Mb region.

Thus we conclude that the results of analysis of different traits are more dependent on the power of the initial study, the presence of actual signals and the polygenic architecture of the trait. Therefore, the improvement that was reported for the height GWAS has not been observed either in BMI GWAS or SCZ GWAS.

**SUPPLEMENTARY TABLES**

**Supplementary Table 1 (separate supplementary Excel file).** Comparison of significant markers from CLUMP statistics identified in the PGC-SCZ set (Purcell et al., 2007; Ripke et al., 2014) and 85 significant independent SNPs identified by conditional and joint analysis (cojo-GCTA) with a threshold of 10^-7^ using the merged Norwegian and German cohorts as the LD reference sample.

Columns A to H show the data from PGC-SCZ CLUMP statistics. Rank 1: order in CLUMP, based on p-value; SNP: reference ID of the SNP according to dbSNP (Sherry et al., 2001); Chr: chromosome; A1: reference allele; A2: second allele; HG19pos: position of the SNP in reference sequence build hg19; Dist: distance between this SNP and the previous SNP (i.e. in the row above); CLUMP p-value: P-value obtained from the PGC-SCZ in the discovery sample.

Columns J to X present the SNPs that were selected in cojo-GCTA and matched to the independent signals from CLUMP. Rank 2: order in cojo-GCTA p-value (if <5x10^-8^); SNP: reference ID of the SNP according to dbSNP (Sherry et al., 2001); Chr: chromosome; HG19pos: position of the SNP in reference sequence build hg19; A1: reference allele; freq: frequency of the reference allele; b: marginal effect; se: standard error of the marginal effect; CLUMP p-value: p-value before cojo-GCTA; bJ: joint effect; bJ_se: standard error of the joint effect; Joint p-value: p-value after cojo-GCTA; Cond. P-value: P-value after conditioning with another SNP in the region; LD if different SNPs: gives the pairwise *r^2^* value between 2 markers if different markers are displayed for CLUMP and cojo-GCTA. The last column gives comments about why some markers lost significance or gained significance after cojo-GCTA.

Rows in blue or green highlight the regions where two signals are defined as independent by CLUMP statistics but are identified as dependent by cojo-GCTA. The two signals are less than 10 Mb apart and are therefore within 10 Mb of the cojo-GCTA sliding window. Within each region, the SNP that was used for conditioning is emphasized in bold.

**Supplementary Table 2 (separate supplementary Excel file).** Markers selected with cojo-GCTA using a threshold below the genome-wide significance level (10^-7^). Rank: order in cojo-GCTA p-value (if >5x10^-8^); SNP: reference ID of the SNP according to dbSNP (Sherry et al., 2001); Chr: chromosome; HG19pos: position of the SNP in reference sequence build hg19; A1: reference allele; freq: frequency of the reference allele; b: marginal effect; se: standard error of the marginal effect; p-value: p-value before cojo-GCTA; bJ: joint effect; bJ_se: standard error of the joint effect; Joint p-value: p-value after cojo-GCTA.

**Supplementary Table 3 (separate supplementary Excel file).** Comparison of significant markers identified in the PGC-SCZ set using CLUMP (Purcell et al., 2007; Ripke et al., 2014) and 101 independent SNPs identified by conditional and joint analysis (cojo-GCTA) with a threshold of 10^-7^ using the merged Norwegian and German cohorts as the LD reference sample for a sliding window of 500 kb (cojo-GCTA-500) or 10 Mb (cojo-GCTA-10M).

Columns A to G show the data from cojo-GCTA-500 statistics. SNP: reference ID of the SNP according to dbSNP (Sherry et al., 2001); Chr: chromosome; HG19pos: position of the SNP in reference sequence build hg19; A1: reference allele; CLUMP p-value: p-value before cojo-GCTA; Joint p-value: p-value after cojo-GCTA; LD_r: pairwise *r^2^* value between 2 markers at the locus.

Columns I to P show the data from PGC-SCZ CLUMP statistics. Rank 1: order in CLUMP, based on p-value; SNP: reference ID of the SNP according to dbSNP (Sherry et al., 2001); Chr: chromosome; A1: reference allele; A2: second allele; HG19pos: position of the SNP in reference sequence build hg19; Dist: distance between this SNP and the previous SNP (i.e. in the row above); CLUMP p-value: p-value obtained from the PGC-SCZ in the discovery sample.

Columns R to AB show the SNPs that were selected in cojo-GCTA-10M and matched to the independent signals from CLUMP. Rank 2: order in cojo-GCTA p-value (if <5x10^-8^); SNP: reference ID of the SNP according to dbSNP (Sherry et al., 2001); Chr: chromosome; HG19pos: position of the SNP in reference sequence build hg19; A1: reference allele; freq: frequency of the reference allele; b: marginal effect; CLUMP p-value: p-value before cojo-GCTA; bJ: joint effect; Joint p-value: p-value after cojo-GCTA; LD if different SNPs: gives the pairwise *r^2^* value between 2 markers if different markers are displayed for CLUMP and cojo-GCTA.

Rows in blue or green highlight the regions where two signals are defined as independent by CLUMP statistics but are identified as dependent by cojo-GCTA with different windows. Within each region, the leading SNP is emphasized in bold.

**Supplementary Table 4 (next page).** Traits and disorders that are associated with the selected genes.

| **Locus** | **Reference** | **Trait** |
| --- | --- | --- |
| *LOC102723362* | Mosing M. et. al. (PMID: 20707712) A genome-wide association study of self-rated health. Twin Res Hum Genet. 2010;13(4):398-403  Lettre G. et. al. (PMID: 21347282) Genome-wide association study of coronary heart disease and its risk factors in 8,090 African Americans: The NHLBI CARe Project. PLoS Genet. 2011; 7(2):e1001300 | Self-rated health  LDL cholesterol |
| *C2orf16, ZNF512, CCDC121, GPN1, SUPT7L, SLC4A1AP, MRPL33, RBKS. BRE, MIR4263* | Weissglas-Volkov D (PMID: 23505323) Genomic study in Mexicans identifies a new locus for triglycerides and refines European lipid loci. J Med Genet. 2013  O'Seaghdha CM (PMID: 20705733) Common variants in the calcium-sensing receptor gene are associated with total serum calcium levels. Hum Mol Genet, 2010  Coram MA (PMID: 23726366) Genome-wide characterization of shared and distinct genetic components that influence blood lipid levels in ethnically diverse human populations. Am J Hum Genet, 2013  Franceschini N (PMID: 23022100) Discovery and fine mapping of serum protein loci through transethnic meta-analysis. Am J Hum Genet, 2012  Kraja AT (PMID: 21386085) A bivariate genome-wide approach to metabolic syndrome: STAMPEED consortium. Diabetes, 2011  Logue MW (PMID: 22159054) A comprehensive genetic association study of Alzheimer disease in African Americans. Arch Neurol, 2011  Manning AK (PMID: 22581228) A genome-wide approach accounting for body mass index identifies genetic variants influencing fasting glycemic traits and insulin resistance. Nat Genet, 2012  Jostins L (PMID: 23128233) Host-microbe interactions have shaped the genetic architecture of inflammatory bowel disease. Nature, 2012 | Hypertriglyceridemia  Calcium levels  Triglycerides  Serum albumin level  Waist Circumference – Triglycerides  Alzheimer's disease  Fasting glucose-related traits (interaction with BMI)  Inflammatory bowel disease |
| *NR2F2, NR2F2-AS1* | Anttila V. (PMID: 23793025) Genome-wide meta-analysis identifies new susceptibility loci for migraine. Nat Genet, 2013  Hromatka B. (PMID: 25628336) Genetic variants associated with motion sickness point to roles for inner ear development, neurological processes and glucose homeostasis. Hum. Mol. Genet., 2015  Kang S. (PMID: 22554406) Family-based genome-wide association study of frontal θ oscillations identifies potassium channel gene KCNJ6. Genes Brain Behav, 2012 | Migraine  Motion sickness  Electroencephalographic traits in alcoholism |

**Supplementary Table 5 (separate supplementary Excel file)**. Comparison of markers from multi-SNP analysis and 96 independent SNPs identified by cojo-GCTA with a threshold of 10^-7^ using the merged Norwegian and German cohorts as the reference LD sample.

Columns A to F correspond to the data from PGC-SCZ statistics (Ripke et al., 2014). SNP: reference ID of the SNP according to dbSNP (Sherry et al., 2001); Chr: chromosome; HG19pos: position of the SNP in reference sequence build hg19; A1: reference allele; A2: second allele; p_value: p-value obtained from the PGC-SCZ in the discovery sample (Ripke et al., 2014).

The columns from H to Q show the SNPs that were selected in cojo-GCTA and matched to the signals which were selected in Multi-SNP. SNP: reference ID of the SNP according to dbSNP (Sherry et al., 2001); Chr: chromosome; HG19pos: position of the SNP in reference sequence build hg19; A1: reference allele; freq: frequency of the reference allele; p-value: p-value before cojo-GCTA; pJ: joint p-value after cojo-GCTA; distance: gives the pairwise distance (in kb) between 2 markers if different markers are displayed for Multi-SNP and cojo-GCTA; *r*^2^ between these SNPs: pairwise *r*^2^ value between 2 markers if different markers are displayed for Multi-SNP and cojo-GCTA. The last column gives comments about why some markers were not selected in the Multi-SNP analysis.

The SNPs in column A were selected by the Multi-SNP tool as top markers for the joint model at the threshold of 10^-7^. However, column F contains corresponding p-values from the PGC-SCZ study.

Rows in purple highlight the signals which were identified as dependent signals by cojo-GCTA, but which did not reach the threshold in Multi-SNP. The SNPs that were in the same 1 Mb window as the ones selected in Multi-SNP with low LD values are highlighted in green.

**SUPPLEMENTARY FIGURES**

**Supplementary Figure 1.** Pipeline of analysis performed for Table 1.

For each different threshold, we run cojo-GCTA analysis. At the first stage, the stepwise procedure is performed on SNPs below a certain threshold using the Norwegian cohort as the reference sample. The selected list of markers with their effects was replicated by a joint analysis using the German cohort as the LD reference sample. We considered a marker as validated for joint effect if it passed the genome-wide significance threshold (5$\times$10^-8^) after the stepwise procedure with the Norwegian LD reference sample and after joint analysis with the German LD reference. The validation criterion was consistency in joint effects and joint p-values within German and Norwegian samples as the reference for LD estimation.

Number of SNPs in discovery statistics below a certain threshold

For example, with threshold 10^-4^ 35,630

Number of SNPs below a certain threshold after stepwise procedure in cojoGCTA with Norwegian sample as reference cohort

For example, with threshold 10^-4^ 1,048

Number of SNPs mapped with German cohort as reference in joint analysis For example, with threshold 10^-4^

1,048

Number of SNPs below 5x10^-8^ after CR

For example, with threshold 10^-4^

186

Number of SNPs below 5x10^-8^ in joint model with German cohort 84

Number of SNPs below 5x10^-8^ with Norwegian and German cohorts as reference and validated as with

–log(p_G_)/-log(p_N_)<2

For example, with threshold 10^-4^

69

Number of SNPs identified in the joint model that did not passed GWSL before CR

For example, with threshold 10^-4^

12

**Supplementary Figure 2.** Allele frequencies estimated in the Norwegian (a) and German (b) cohorts against the allele frequencies used in the summary file for the 83 validated markers at the threshold 10^-7^. Allele frequencies estimated in the merged Norwegian and German cohorts (c) vs the frequencies used in the summary statistics for 85 significant SNPs at the threshold 10^-7^. The 81 SNPs that matched the clumped significant markers are in black. The four SNPs that did not are in green.

**b.**

**a.**


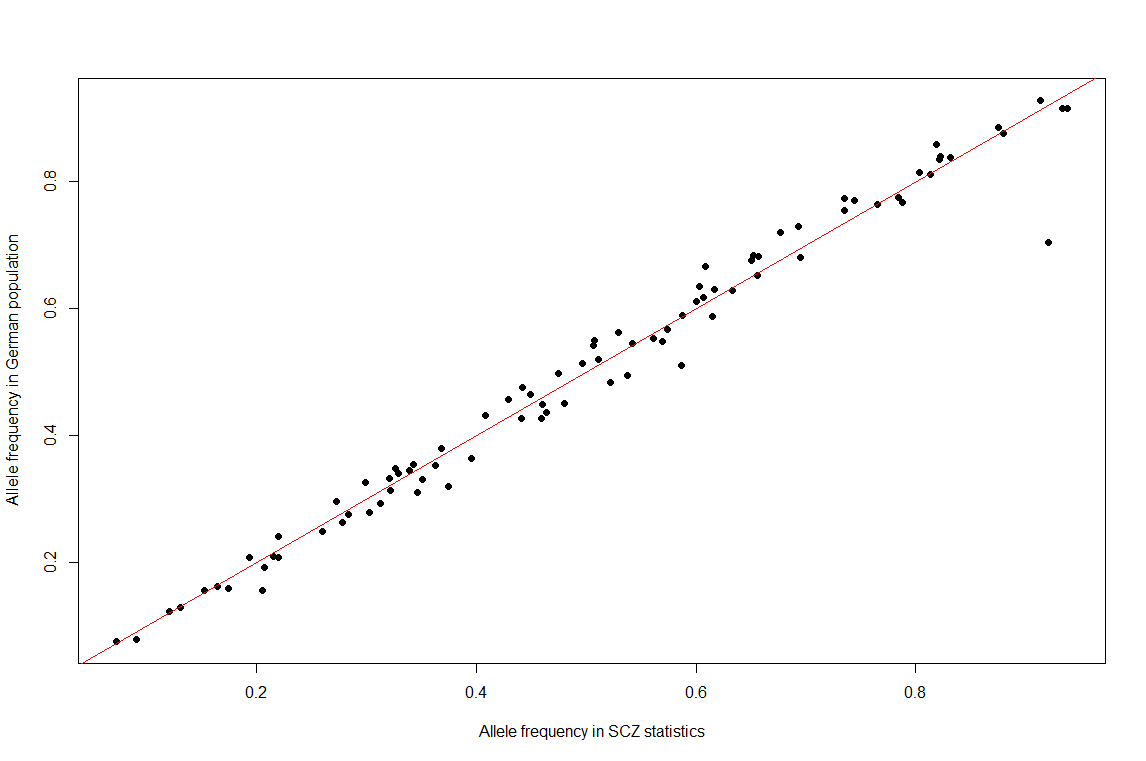

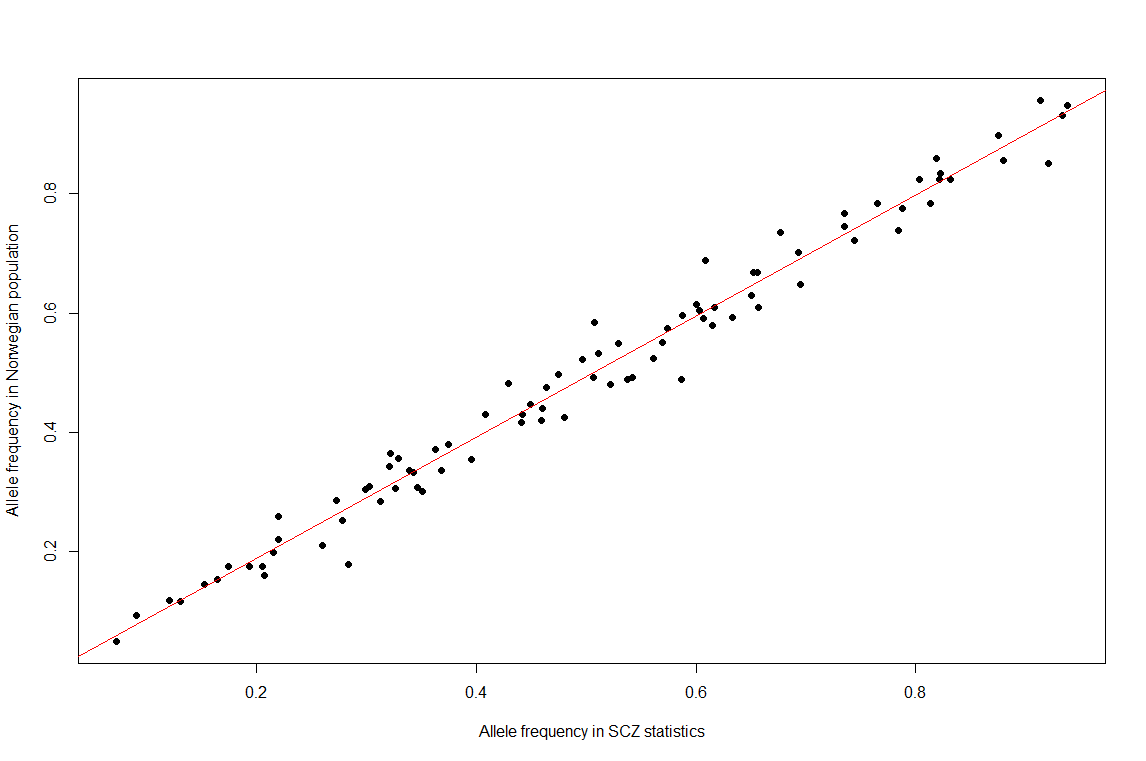


*r^2^*=0.985

*r^2^*=0.987

**c.**


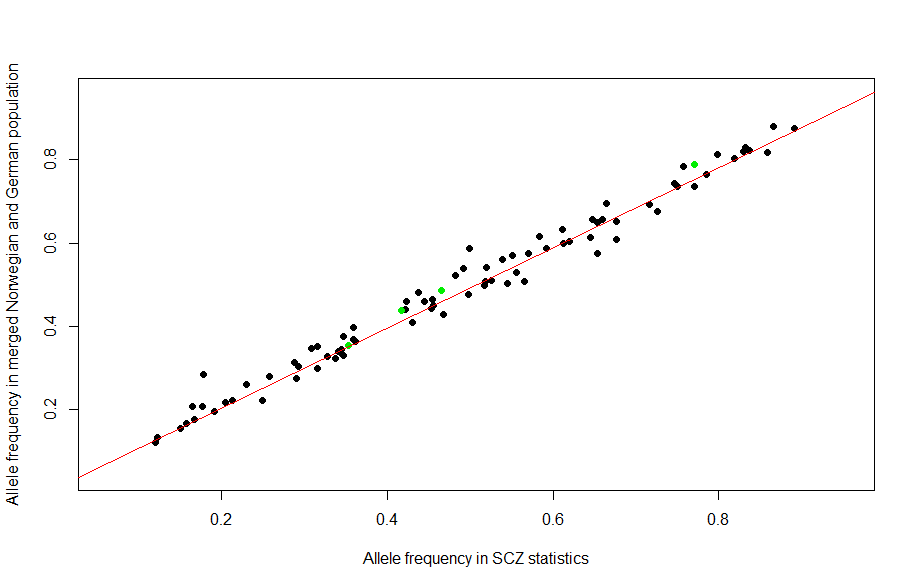


*r^2^*=0.987

**Supplementary Figure 3.** Multidimensional scaling analysis of population structure.

Multi-dimensional scaling analysis of the Norwegian (TOP, NCNG) and German (Sample3-Quad, Sample4-H, Sample5_B) samples and HapMap3 populations. (a) A scatterplot of the first three principal components of the Norwegian and German samples is overlaid with 11 distinct HapMap3 populations (MKK, LWK, ASW, GIH, MEX, CHD, JPT, TSI, YRI, CHB, CEU). The majority of the German and Norwegian samples cluster within the European (EUR, TSI) HapMap subsamples. (b) The European populations in detail: Sample5_B (HNR-HumanOmniExpress_12v1_B), Sample4_H (HNR-HumanOmniExpress_12v1_H), Sample3_Quad (HNR-HumanOmni1-Quad_v1_H), Norwegian samples (TOP and NCNG), Toscanian (TSI) and Central-European sample (CEU).

**a.**
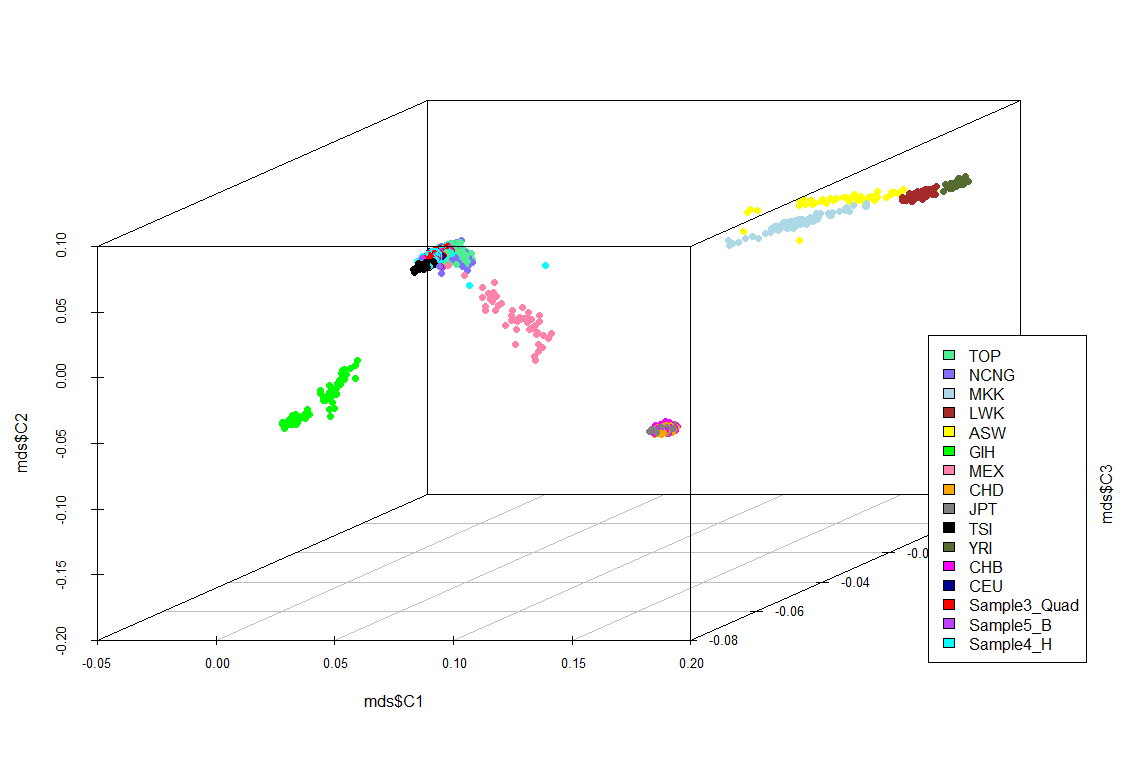


**b.**
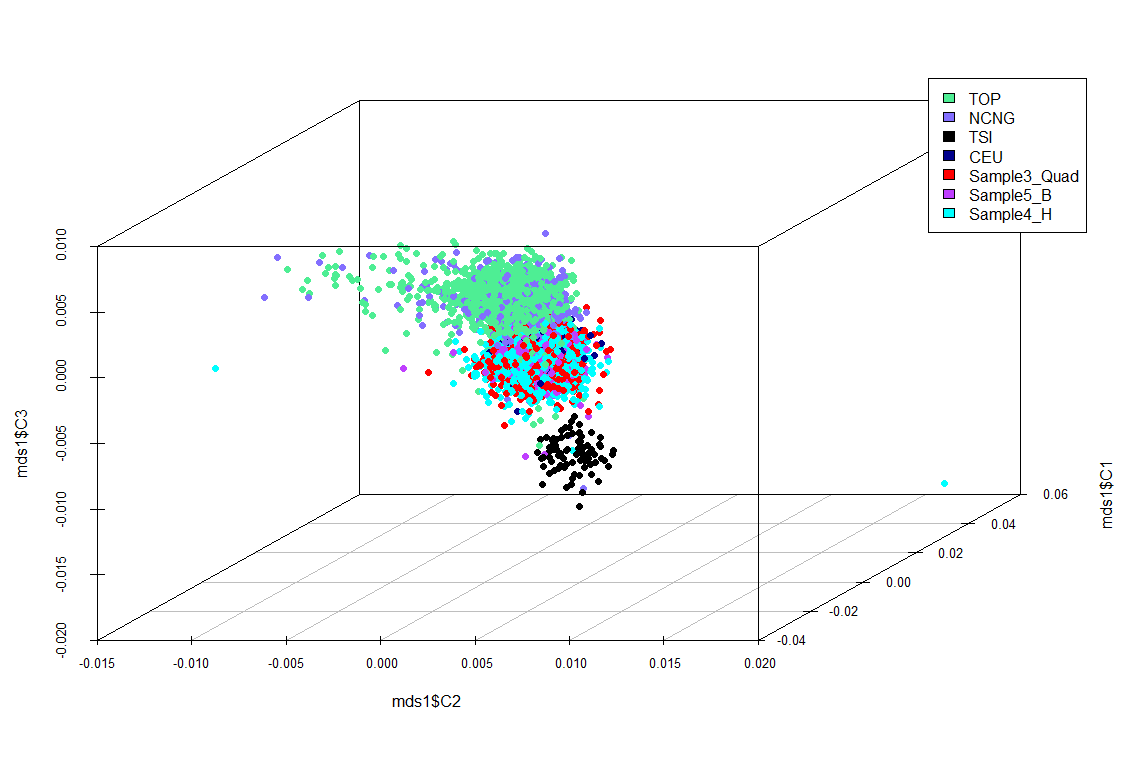


**SUPPLEMENTARY REFERENCES**

Abecasis, G., & Li, Y. (2007). MACH 1.0. Retrieved from sg.sph.umich.edu//abecasis/MaCH/

Athanasiu, L., Mattingsdal, M., Kähler, A. K., et al. (2010). Gene variants associated with schizophrenia in a Norwegian genome-wide study are replicated in a large European cohort. *J Psychiatr Res*, *44*, 748–753.

de Moor, M. H. M., van den Berg, S. M., Verweij, K. J. H., et al. (2015). Genome-wide association study identifies novel locus for neuroticism and shows polygenic association with Major Depressive Disorder. *JAMA Psychiatry*, *72*, 642–650.

Espeseth, T., Christoforou, A., Lundervold, A. J., et al. (2012). Imaging and Cognitive Genetics: The Norwegian Cognitive NeuroGenetics Sample. *Twin Research and Human Genetics*, *15*, 442–452.

Manning, A. K., Hivert, M.-F., Scott, R. A., et al. (2012). A genome-wide approach accounting for body mass index identifies genetic variants influencing fasting glycemic traits and insulin resistance. *Nature Genetics*, *44*, 659–669.

Morris, A. P., Voight, B. F., Teslovich, T. M., et al. (2012). Large-scale association analysis provides insights into the genetic architecture and pathophysiology of type 2 diabetes. *Nat Genet*, *44*(9), 981–990.

Nikpay, M., Goel, A., Won, H.-H., et al.(2015). A comprehensive 1,000 Genomes-based genome-wide association meta-analysis of coronary artery disease. *Nature Genetics*, *47*, 1121–30.

Purcell, S., Neale, B., Todd-Brown, K., et al. (2007). PLINK: a tool set for whole-genome association and population-based linkage analyses. *American Journal of Human Genetics*, *81*(3), 559–75.

Ripke, S., Neale, B. M., Corvin, A., et al. (2014). Biological insights from 108 schizophrenia-associated genetic loci. *Nature*, *511*, 421–427.

Schmermund, A., Möhlenkamp, S., Stang, A., et al. (2002). Assessment of clinically silent atherosclerotic disease and established and novel risk factors for predicting myocardial infarction and cardiac death in healthy middle-aged subjects: Rationale and design of the Heinz Nixdorf RECALL Study. *American Heart Journal*, *144*, 212–218.

Sherry, S., Ward, M., Kholodov, M., et al. (2001). dbSNP: the NCBI database of genetic variation. *Nucleic Acids Research*, *29*, 308–11.

Stein, J. L., Medland, S. E., Vasquez, A. A., et al. (2012). Identification of common variants associated with human hippocampal and intracranial volumes. *Nature Genetics*, *44*, 552–561.

The Enhancing Neuroimaging Genetics through MetaAnalysis (ENIGMA) Consortium. ENIGMA2 1KGP Cookbook (v3). Retrieved from http://enigma.ini.usc.edu/wp-content/uploads/2012/

van den Berg, S. M., de Moor, M. H. M., McGue, M., et al. (2014). Harmonization of Neuroticism and Extraversion phenotypes across inventories and cohorts in the Genetics of Personality Consortium: an application of Item Response Theory. *Behavior Genetics*, *44*, 295–313.

Yang, J., Ferreira, T., Morris, A. P., Medland, S. E., et al. (2012). Conditional and joint multiple-SNP analysis of GWAS summary statistics identifies additional variants influencing complex traits. *Nature Genetics*, *44*, 369-375.
